# Judo training program improves brain and muscle function and elevates the peripheral BDNF concentration among the elderly

**DOI:** 10.1038/s41598-022-17719-6

**Published:** 2022-08-16

**Authors:** Sylwester Kujach, Maciej Chroboczek, Joanna Jaworska, Angelika Sawicka, Miroslaw Smaruj, Pawel Winklewski, Radoslaw Laskowski

**Affiliations:** 1 Department of Human Physiology, Medical University of Gdańsk, Gdańsk, Poland; 2 Department of Physiology, Gdansk University of Physical Education and Sport, Gdańsk, Poland; 3 Department of Physiology, Medical University of Gdańsk, Gdańsk, Poland; 4 Applied Cognitive Neuroscience Lab, Department of Human Physiology, Medical University of Gdańsk, Gdańsk, Poland; 5 Department of Theory of Sport and Human Motorics, Gdansk University of Physical Education and Sport, Gdańsk, Poland; 6 2nd Department of Radiology, Medical University of Gdańsk, Gdańsk, Poland

**Keywords:** Physiology, Neuroscience

## Abstract

Programmed exercise interventions modulating both physical fitness and cognitive functions have become a promising tool to support healthy aging. The aim of this experiment was to determine the effect of a 12-week judo training (JEX) on cognitive processing and muscle function among the elderly. Forty participants were divided into two groups: the JEX group and the control group (CTL). Before and after 12-week of JEX, participants performed a battery of physiological and psychological tests. The peripheral level of brain-derived neurotrophic factor (BDNF) was analyzed. A 12-week JEX intervention led to improved Stroop performance reflected by a shortening of the response time related to Stroop “naming” interference. In addition, the peripheral concentration of BDNF was significantly increased following the JEX compared with the CTL group. In response to JEX, balance and lower limb strength significantly increased. The current results suggest that JEX could have beneficial effects on cognitive functions, denoted by elevated peripheral BDNF, as well as on balance and strength abilities. A combination of positive effects with respect to movement and cognition makes JEX an ideal preventive lifestyle modification for the aging population.

## Introduction

Normal aging entails cognitive decline, represented as dysfunction in working and long-term memory, processing speed, and inhibition, as well as slow and continuous loss of muscle mass—a process defined as ‘sarcopenia’—that contributes to diminished muscle strength and mobility^1–3^. Therefore, aging, as well as neurodegenerative diseases, are also associated with impairments in movement and various cognitive domains^1,4^.

Physical activity remains the most effective lifestyle modification to counteract brain and muscle senescence, preventing inflammation and the development of neurodegenerative processes^5,6^. Staying physically active slows down the negative age-dependent physical fitness and cognitive changes^7^. Older people who have been active throughout their lives are at a reduced risk of developing metabolic and neurodegenerative diseases in relation to their sedentary counterparts^4,6,8,9^.

Most physical training interventions applied among the elderly population are based on low to moderate aerobic exercise intensity protocols^4^. While vigorous exercise can activate the hypothalamic–pituitary–adrenal axis inducing psychophysical stress, it is assumed that low to moderate exercise is safe for older people and does not induce psychological or physiological stress response^4,10^. On the other hand, regarding the muscle adaptation paradigm, low-intensity exercise neither induces beneficial adaptive changes (e.g., aerobic/anaerobic capacity improvement) nor prevents muscle mass decline during the aging process^11^. Therefore, effective types and a suitable intensity of exercise are still being sought^4,9,12,13^. It has also been shown that chronic exercise had a great effect on the performance of executive function tasks, which are considered higher‐order cognitive functions controlled by frontal brain areas^8^. Moreover, physical activity programs characterized as multi-modal forms of exercises that incorporate aerobic exercise, anaerobic/resistance exercise, coordinative exercise, and performed with variable intensity could be a powerful stimulant to improve cognitive function^14,15^.

Martial arts, because of their comprehensive focus on movement and sensorimotor control, are prime examples of physical exercises that could help to keep the aging population fit and healthy^6^. The martial arts are often classified as “hard” or “external” (e.g., judo, karate, taekwondo, and kung fu) and “soft” or “internal” (e.g., tai-chi and health-qigong)^16^. “Soft” martial arts are characterized by relaxed, smooth movements often performed slowly, targeting the control of posture during the movements executions^16,17^, whereas “hard” are characterized by fast, vigorous, and dynamic movements, relying on physical strength, speed, and endurance^18^. As a “hard” martial art, Judo demands several specific characteristics, such as correct posture, balance, strength, velocity, and power^18–20^. It has been also shown Judo requires higher levels of muscle strength and power, for applying the throws, holding grips, or locking^21^. While, the maintenance and proper execution of the intermittent exercises, as well as the recovery process during the rest periods, are mainly supported by aerobic metabolism^21^. Thus, judo participants have shown elevated anaerobic and aerobic capacity^18,21^. Moreover, judo training programs include safe fall techniques that can protect against injuries^19,22^. The fall rate among the elderly is frequently a direct consequence of diminishing physical function, whereas exercise programs focusing on strength and balance, such as judo, reduce falls and fear of falling in older age groups^22^. Judo techniques should also be performed with accuracy, a factor that stimulates motor cortex and sensorimotor control mechanisms^18,19,22^. Additionally, judo exercise could involve more cognitive loads and demands than other so-called “closed skills exercise”, like walking, jogging, or swimming. In contrast to “closed skills” activities where exercises are performed in a predictable and stable environment in which participants are less likely to be exposed to multisensory stimuli^23^, judo training as “open-skills” requires to adapt to a continually changing environment^23,24^. Furthermore, “soft” as well as “hard” martial arts, including Judo, requires participants to perform a sequence of movements in order to shape the technique (uchikomi), also the judo practitioner should correctly read the partner’s intentions to facilitate his movement (throwing or grappling)^18,19^. Also, training fighting (randori) needs quick reaction time and persistence of attention. Thus, Judo training could develop both physical performances as well as cognitive functions such as executive functions (for example: selective visual attention or cognitive flexibility), processing speed as well as working memory and learning^17^. It is worth adding that judo practitioners (more than 10 years of judo experience) possessed higher gray matter volume in various regions of the brain associated with motor learning, planning, and execution, as well as memory and cognitive processes when compared with healthy controls. It was speculated that these adaptations were the result of the complex motor skills required during judo training^25^. Practicing judo techniques also requires cooperation with a partner (uke), what is more, the judo sessions are conducted in groups of participants mainly. Consequently, these factors can strengthen social interactions, positively influencing the cognitive function of older people^26^.

Therefore, engagement in a judo training program could be an appropriate tool not only to improve muscle function but also mental health.

In general physical exercise can induce beneficial changes in the brain via increased arousal, including augmented concentrations of noradrenaline and dopamine, elevated cerebral blood flow as well as modulation of brain metabolism and neuroprotective protein synthesis^9,27,28^. The key protein that can modulate cognitive functions is a brain-derived neurotrophic factor (BDNF)^9^. BDNF plays an important role in memory formation, synaptic plasticity, neurogenesis, and increased brain connectivity^9,12,27^. The peripheral BDNF concentration can be modulated via acute exercise^29,30^, whereas the effects of chronic exercise interventions are ambiguous^31,32^. A post-exercise peripheral BDNF increase has been associated with improvement in human cognitive function^29^. It is worth mentioning that 80% of the peripheral BDNF comes from the brain, while 20% may arise from peripheral tissues, where physical exercise is a major releasing stimulus^33,34^. To the best of our knowledge, no studies have evaluated the effect of a judo-based exercise program on both cognitive function and physical fitness among the elderly. Therefore, the main purpose of the present study was to investigate whether 3 months of Judo training program (JEX) affects the cognitive processes, physical functions, and the peripheral level of BDNF.

## Results

### Body composition

At baseline, there were no significant differences in anthropometric characteristics between the groups (Table 1).

**Table 1:** Demographic and clinical characteristics.

Variables	JEX (n = 20)		CTL (n = 20)	
	Pre	Post	Pre	Post
Age (yr)	67.5 ± 5.3	67.9 ± 5.3	67.6 ± 5.1	68.0 ± 5.1
Height (cm)	168.1 ± 3.2	168.1 ± 3.2	169.1 ± 4.1	169.1 ± 4.1
Weight (kg)	69.3 ± 15.0	68.2 ± 14.6	72.2 ± 12.6	71.1 ± 12.4
FAT (%)	32.1 ± 6.7	32.1 ± 6.4	34.3 ± 5.3	33.8 ± 5.3
BMI (kg·m^−2^)	24.4 ± 2.6	24.2 ± 2.5	25.1 ± 2.5	24.7 ± 2.4

Values are means ± SD. *JEX* judo group, *CTL* control group.

### Cognitive functions

There were no differences between the JEX and CTL groups in the performance of the Stroop ‘naming’ and ‘reading’ interference for the pre-sessions. Based on a repeated-measured two-way ANOVA, there was a significant group (JEX/CTL) × time (PRE/POST) interaction for the ‘naming’ interference execution time (F(1,38) = 4.20 p < 0.05, η^2^ = 0.10). Moreover, post hoc Bonferroni test analysis revealed a significant decrease in the Stroop “naming” interference execution time in the JEX group (pre vs. post-training intervention) (p < 0.01) (Table 2). Neither the group main effect nor the group × time interaction were significant in the ‘reading’ interference execution time as well as error rate in both conditions (Table 2).

**Table 2:** The execution time and error rate for the Stroop ‘naming’ and ‘readings’ interference in response to 12 weeks of the judo exercise training program.

Variables	JEX		CTL	
	Pre	Post	Pre	Post
Stroop ‘naming’ Interference RT (ms)^b^	0.27 ± 0.04	0.12 ± 0.01^a^	0.28 ± 0.04	0.23 ± 0.04
Stroop ‘reading’ Interference RT (ms)	0.11 ± 0.02	0.12 ± 0.03	0.11 ± 0.01	0.14 ± 0.01
Stroop ‘naming’ Interference ER (%)	1.37 ± 0.50	1.05 ± 0.32	1.25 ± 0.27	1.41 ± 0.32
Stroop ‘reading’ Interference ER (%)	0.94 ± 0.46	0.74 ± 0.20	1.05 ± 0.29	1.02 ± 0.16

Values are mean ± SEM. *JEX* judo exercising group, *CTL* control group, *RT* reaction time, *ER* error rate.

^a^Significant differences from pre-JEX, p < 0.01.

^b^Significant group (JEX/CTL) × time (PRE/POST) interaction, p < 0.05.

### Effect of JEX on the peripheral BDNF concentration

**Figure 1:**
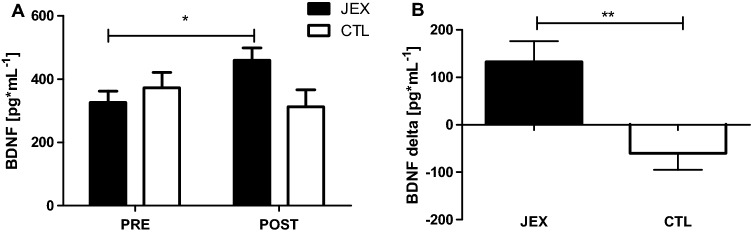
The effect of JEX on the peripheral BDNF concentration (**A**); and the contrast between the JEX delta (post–pre) and CTL delta (post–pre) (**B**). The values represent the mean. The error bars indicate the standard error of the mean. JEX pre vs. post-intervention *p < 0.01 post hoc Bonferroni test (**A**), contrast between the JEX delta and CTL delta **p < 0.001 Mann–Whitney *U* test (**B**).

Analyzing the effect of JEX on the peripheral BDNF concentration, a two-way repeated-measures ANOVA with post hoc Bonferroni tests revealed a significant group × time interaction (F(1,38) = 11.85, p < 0.01, η^2^ = 0.23; Fig. 1A). Moreover, there were significant differences between the JEX and CTL groups in the BDNF delta concentration (U = 66.0, p < 0.001; Fig. 1B).

### Muscle functions

#### Postural control

**Figure 2:**
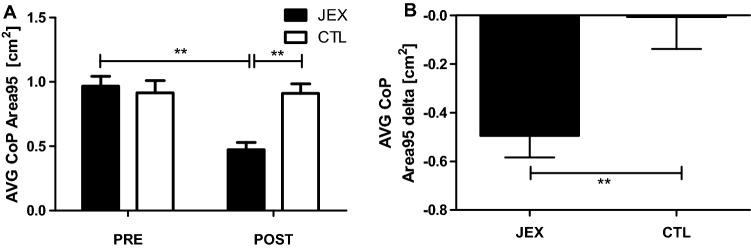
The effect of JEX on postural control, namely AVG CoP Area 95 (**A**), and the contrast between the JEX delta (post–pre) and the CTL delta (post–pre) (**B**). The values represent the mean. The error bars indicate the standard error of the mean. JEX pre vs. post-intervention and post-JEX vs. post CTL **p < 0.001 post hoc Bonferroni test (**A**), contrast between the JEX delta and CTL delta **p < 0.001 Mann–Whitney *U* test (**B**).

Based on a repeated measured two-way ANOVA with post hoc Bonferroni tests, the postural control data showed a significant group × time interaction for AVG CoP Area95 (F(1,38) = 9.27, p < 0.01, η^2^ = 0.19; Fig. 2A). There was also a significant difference between the JEX and CTL groups for AVGCoP Area95 delta (U = 72.0, p < 0.001), indicating a beneficial effect of JEX on the balance parameter (Fig. 2B).

#### Muscle strength

**Figure 3:**
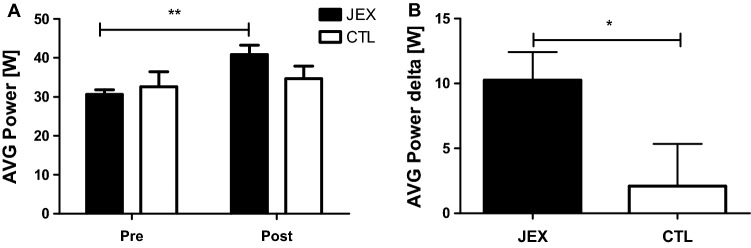
The effect of JEX on AVG power (**A**), and the contrast between the JEX delta (post–pre) and the CTL delta (post–pre) (**B**). The values represent the mean. The error bars indicate the standard error of the mean. JEX pre vs. post-intervention **p < 0.01 post hoc Bonferroni test (**A**) and contrast between the JEX delta and CTL delta *p < 0.05 Mann–Whitney *U* test (**B**).

The ANOVA for the average power demonstrated a significant group × time interaction (F(1,38) = 4.38, p < 0.05, η^2^ = 0.19; Fig. 3A). Post hoc Bonferroni tests revealed a significant increase in the average power (AVG power, p < 0.01) in the JEX group after the training program (Fig. 3A). Moreover, the AVG power delta was significantly different between the JEX and CTL groups (U = 116, p < 0.05; Fig. 3B).

## Discussion

There are several new findings from the study. Specifically, 12 weeks of JEX resulted in (1) improvements in executive functions, (2) augmentation of postural control and muscle strength, (3) increased peripheral concentrations of neuroprotective BDNF.

In the present study, we illustrated that chronic JEX intervention enhances cognitive functions in older adults, as reflected in the Stroop ‘naming’ interference performance. This result is consistent with previous studies that have demonstrated chronic exercise enhances executive functions in older adults^7^. Additionally, Alves et al.^35^ reported executive function improvements (Stroop test) in response to high-intensity interval training compared to stretching exercises. Judo training can be considered a type of interval training as exercises are interspersed with rest periods during a single training unit^35^. Cognitive benefits have also been reported in response to yoga and tai chi exercise programs; these benefits were significantly larger than those observed for aerobic exercise interventions^6^. These physical exercise modalities are sometimes categorized as mind–body exercises and have been shown to impact different aspects of physical abilities (e.g. balance, flexibility, and agility) similarly to judo, and in comparison to typical aerobic exercise^6^. Very recently, Ludyga et al. observed that the judo training program elicited benefits for response inhibition in preadolescent children^36^. Moreover, researchers have proposed that there is a meaningful relationship between response inhibition in the Stroop Colour and Word Test and driving performance^37^. Thus, the effectiveness of JEX training in this cognitive domain can directly translate into the everyday functioning of the elderly. Interestingly, Douris et al.^38^ demonstrate that the Korean martial art classes (Soo Bahk Do) were effective in executive functions improvement possibly because increased cortical recruitment is necessary for the complex, coordinated motor demands of the martial art compared to the more repetitive actions of walking. Moreover, they concluded the more repetitive actions of walking did not offer the same cortical stimulation and therefore only afforded improvements in attention and processing speed, but not executive function^38^. Recent animal and human studies have revealed that exercise enhances cognition via neurotrophins and catecholamine production, which is known to mediate neural plasticity and energy metabolism in the brain^9^. It has been also shown that physical training benefits functional connectivity in the medial and lateral temporal cortices^12^. Furthermore, exercise-induced neurovascular adaptations in the hippocampus have been associated with cognitive function^39^. Moreover, rodents that participated in motor-skill learning tasks (comparable to coordination training) showed a greater number of synapses per neuron, substantially increased the volume of the molecular layer per Purkinje neuron, and also sufficiently increased the number of capillaries. Further, they had significantly more parallel fiber to Purkinje cell synapses than walking and inactive animals^40^. Since Judo training requires also perceptual and higher-level cognitive processes, such as attention, and adaptive aspects of postural control or coordination, might facilitate cognitive processing by increasing the number of synapses per neuron in the required brain areas. Thus post-training adaptation in brain metabolism and improvement of brain connectivity may have contributed to the reduction in Stroop task reaction time among the JEX group.

Also, there could be post-training cognitive facilitation due to the release of various neurotransmitters from several neuromodulatory systems such as ascending projections to the prefrontal cortex, a structure that is critical for cognitive functioning^41^. Animal studies have revealed that exercise leads to increased release of acetylcholine from the nucleus basalis, basal levels of noradrenaline in the locus coeruleus, and dopamine (DA) release in the nucleus accumbens^28^. Interestingly, DA was upregulated in rodent brains subjected to 8 weeks of running-wheel exercise and the binding affinity between DA and the DA receptor was also increased by physical training. Moreover, running-wheel exercise increased cortical levels of DA (see Lin and Kuo^41^ for review). Altogether, an increasing number of animal and human studies have confirmed that exercise modulates several neurotransmitter systems influencing cognitive functions, although we did not replicate the role of neurotransmitter systems in the current JEX study. For the first time, we demonstrated that 12 weeks of JEX improves Stroop interference performance in older adults. Therefore, we postulate that the current JEX chronic intervention is beneficial to executive functions among the elderly.

We also found that 12 weeks of JEX improved muscle strength and postural control. In older adults, the maintenance of muscle strength is crucial for daily living activities (e.g., bending, lifting, reaching, walking), preserving lean body tissue to prevent obesity, and improving glucose utilization and/or counteracting bone loss^42^. Our data are in agreement with previous studies demonstrating upper and lower body strength increases following a 4-month judo training (2 × 60 min sessions per week) among the elderly^42^. Further, a study by Arkkukangas et al.^22^ demonstrated post judo training improvement in physical functions (Short Physical Performance Battery test-SPPBt) and falling techniques compared to a control group of working-age adults^22^. Similarly, a 12-week taichi intervention facilitated muscle function, denoted by increased walking speed and performance in the SPPB test^43^. Moreover, 3 months of taekkyon, a Korean form of martial arts, improved functional mobility, balance, and lower extremity strength in older women, similarly to the tai chi training^44^. Therefore, martial arts such as tai chi, taekkyon, or judo, where many basic exercises and movements are based on obtaining balance, could be a strong stimulus shaping this ability. Moreover, the correct judo throw execution requires the disturbance of the partner’s balance (kuzushi), which may lead to an overthrow of him. A partner with a disturbed balance tries to correct it, thereby developing this ability^16,19^. Hence, judo practitioners develop the ability to balance even more effectively. Similarly to our observation, were highlighted among hard martial arts practitioners^16^. Aside from known relationships between strength balance deficits and falls, one omitted aspect is being able to or learning to get up and down from the floor and falling safely^22,45^. It is worth mentioning that there are also safe falling techniques (ukemi) in the judo exercises^19,22^. Thus, judo training protects in two ways: it helps develop strength and balance to prevent falls and, in the event of a fall, it makes it safer and ideally non-injurious^18,22^. The strength and balance improvement, as well as the safe falling ability in response to judo training, suggest judo is an excellent strategy to prevent injuries due to falls.

It is well known that movement deterioration and postural instability are defining features of neurodegenerative disorders, including Parkinson disease (PD)^46^. Many researchers have indicated improvements in muscle strength and overall postural balance in patients with PD undergoing various exercise regimes^46^. The risk of PD increases with age, and training programs focused on movement control and balance, such as Judo, could provide a non-pharmacological prevention tool. Similar findings to those in this study have been reported in taekwondo beginners^47^.

To the best of our knowledge, no studies have associated changes in peripheral BDNF after JEX training in the elderly. We found that the 12 weeks of JEX increased the peripheral BDNF concentration. Acute as well as chronic physical exercise may lead to an increase in the BDNF concentration^29,32^. These exercise-related increases may support a reduction in mood disorders and the protection and regeneration of various tissues, resulting in the facilitation of cognitive function^27,29,48^. It is worth adding that exercise-related upregulation of BDNF may help to compensate for age-dependent reductions in neurogenesis, synaptogenesis, synaptic plasticity, and learning and memory, leading to a more resilient brain in the context of age-related structural and functional changes^9,12,27,49^. Although we did not observe a relationship between the Stroop test results and peripheral BDNF concentration, we cannot rule out the potential impact of central BDNF level on cognitive processes. Since the circulating BDNF concentration reflects the central concentration of this protein^33^, we postulate that facilitation of executive function could be associated with exercise-related BDNF upregulation. Consequently, JEX can be considered as a preventive lifestyle modification, particularly in relation to the increasing elderly population.

This study is not without limitations. The investigated group consisted mostly of females. Therefore, the results should be interpreted with caution when considering the elderly male population. Nevertheless, the females were all post-menopausal, and therefore the effects of sex hormones can be considered limited. Furthermore, we did not control the subjects’ nutritional habits as well as the mood state, which may also modulate cognitive processes. Although we did not measure the fall frequency, the improvement in balance expressed in the postural control test may be an indirect indicator of a reduced fall rate^50,51^. It should also be noted, that the lack of an exercising control group in the current study limits our ability to conclude that the observed changes were a direct result of the judo program. Thus the future experiment should consider comparing the JEX with a general exercise program (judo vs. aerobic/resistance exercise) to better determine whether these improvements were a result of general physical activity or specifically due to the JEX program. Nonetheless, JEX could certainly be an interesting physical activity alternative for the elderly. Especially, since also covers unique safe fall techniques protecting the elderly from injury. Moreover, it would be also interesting to compare judo training with other types of martial arts (e.g. tai-chi, karate; taekwondo; aikido) to determine whether the type of martial arts training may differentially affect cognition and physical performance.

Future studies, with extensive use of neuroimaging techniques, should focus on the effects of JEX on brain structural and functional connectivity as well as changes in cerebral perfusion and metabolism.

## Conclusions

We revealed that 12 weeks of a JEX training program improved muscle strength and postural control, augmented executive functions, and increased the peripheral BDNF concentration. The combination of positive effects with respect to strength, balance, and cognition makes JEX an ideal preventive lifestyle modification for the aging population. In particular, it could be potentially useful in delaying or decreasing the consequences of aging.

## Methods

### Participants

**Figure 4:**
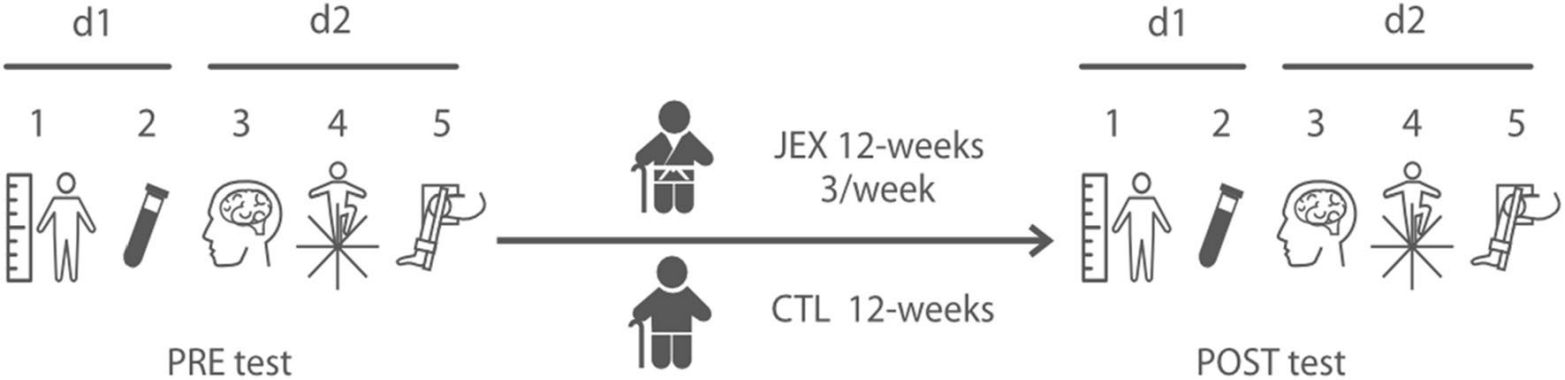
Study protocol: investigational timeline. *1* Anthropometric measurements, *2* Blood sampling, *3* Cognitive testing, *4* Body balance, *5* Muscle strength, *d1* first day, *d2* second day.

Forty-five elderly subjects (38 females and 7 males) participated in the study. The participants were recruited by the City Health Promotion Center and randomly divided into two groups: JEX and control (CTL). The study participants were not involved in any physical activity, and their activity was limited to basic daily activities such as shopping and cleaning. The study was announced by research staff through leaflets, education lectures, and presentations. At the recruitment stage, after open public education lectures (i.e. history of Judo or physical activity health benefits), 45 participants expressed their interest in participating in the study. Five females withdrew from further participation due to personal reasons. Finally, 40 participants (age 67.7 ± 5.2, females n = 33, males = 7) joined the experiment. Written informed consent was obtained from all participants before executing the training protocol. The research was approved by the Bioethical Committee of the Regional Medical Society in Gdansk KB-20/17 and was conducted in accordance with the Declaration of Helsinki. The exclusion criteria included the inability to stand or move independently, the presence of a symptomatic cardiovascular or respiratory disease, a history of myocardial infarction or stroke, reported painful arthritis, spinal stenosis, amputation, painful foot changes or neuropathy, systolic pressure above 160 mmHg or diastolic pressure above 100 mmHg, known arrhythmia or the presence of a pacemaker, Parkinson’s disease (PD), metastatic cancer or immunosuppressive therapy. All participants were characterized by similar psycho-motor abilities levels, additionally, they had no previous experience with Judo practicing. Subjects from the CTL group did not undertake any exercises during the experiment and their physical activity was limited to everyday life functioning. After the entire experiment, all subjects could participate in ongoing judo training. All participants completed the initial assessment. Interpretive lectures, demonstration training as well as familiarisation with laboratory devices were carried out twice, a month as well as a week before the experiment. Three days before and after the entire training intervention, the participants performed a battery of tests in the following order: first day, anthropometric measurements and blood collection; second day, cognitive, body balance, and muscle strength testing. An overview of the experimental procedure is presented in Fig. 4.

### Training intervention

Subjects in the JEX group participated in 36 training sessions for 12 weeks, three times a week. Each training session lasted 45 min. The training program was based on selected exercises from the Kodokan Judo Institute in Tokyo^19^. Training sessions were carried out in a judo training room (dojo) with specialized mattresses (tatami). The instructor informed the participants about the rules of safety and hygienic behavior in judo classes. The participants received regulations and a glossary with the names of judo technical elements. A 12-week training program was supervised by three qualified judo instructors and two master class judo coaches (7th DAN); the latter two managed the training program, which allowed to individualize the training process and control the progress in the motor skills acquisition by the participants. Each training session comprised three sections: warm-up, the main training and cool down. At the beginning of the JEX program, the qualified coaches paid attention to familiarizing the participants with judo technics as well as not exceeding the technic demonstration time during a single judo session. Thus during a single training session, participants learned “step by step” judo technique elements, and the exercise intensity was maintained at a level adjusted to the participant’s safety, ensuring the correct performance of judo techniques. A detailed is provided in the supplementary material.

### Anthropometric measurements

Body mass (BM) and body composition were analyzed by using a multi-frequency impedance body composition analyzer (In Body 720, Biospace, Seoul, Korea). This apparatus accurately analyses body water and body composition, including fat mass, free fat mass, skeletal muscle mass, and soft lean mass^29^.

### Cognitive functions

Cognitive testing was carried out by the neuropsychologist and took a place in the morning hours in an isolated and quiet room, where only the participant and the researcher could stay. During the test, the participant was wearing headphones to minimize the inflow of external stimuli. The current form of the Stroop test is a computerized version of the original color-word interference paradigm by Stroop^52^. Test form S8 (Vienna Test System SCHUHFRIED, Mödling, Austria) applied in this research differentiated between so-called ‘congruent’ items—the color and meaning of the word match—and ‘incongruent’ items–the color and meaning of the word do not match. A color word in red, green, yellow, or blue appeared in the upper third of the screen. Four colored target points were displayed in the lower part of the screen corresponding to four colored buttons on the keyboard. The task was to press the correct respective color button. In the ‘reading’ part, the participant should read the written word not paying attention to the font color (e.g. red—correct answer red), while in the ‘naming’ part should respond to the font color regardless of the written word (e.g. red—correct answer green). The total task time was 10 min, during which, 276 stimuli (including 20 for practice) were shown to participants. The stimulus remained on the screen until the response was given. Corrections to previous items were not possible. After each entry, the next item appeared immediately. All words were written in Polish. In the S8 test form, the words are first presented in the appropriate color as the baseline condition. The main scoring variables are ‘reading interference’ (the difference in reaction times between the reading interference condition and reading baseline) and the ‘naming interference’ (the difference in reaction times between the color naming interference condition and the color naming baseline) as well as error rate. All participants underwent familiarization 1 month before experiments as well as a practice session before performing the task. The cognitive test was the same as in a previously published study^53^.

### Blood sampling and analysis

Blood samples were taken from the antecubital vein into vacutainer tubes before and after the training intervention to evaluate serum concentrations of BDNF. The samples were centrifuged at 2000*g* for 10 min at 4 °C. The separated serum samples were then frozen and kept at − 70 °C until later analysis. The intra-assay coefficients of variability (CVs) and inter-assay CVs reported by the manufacturer were 4–6% and 8–10%, respectively. Serum BDNF was determined via an enzyme immunoassay method using commercial kits (R&D Systems, USA, catalog no. DBD00).

### Postural control assessment

The postural control measurements of all participants were taken in the morning on an AccuGait force platform (Advanced Mechanical Technology Inc., Watertown, MA, USA), recording the displacement of the center of pressure (COP) using the AMTI software. The static postural control in the upright position was based on a protocol that included trials on both legs, a single leg, and the tandem stance. Each trial lasted 30 s, with a frequency sampling of 100 Hz that was low pass filtered at 5 Hz, using a rectangular filter in the frequency domain. The measurements were repeated three times for each trial. During each trial, all patients were monitored by an observer for safety and were asked to stand as still as possible, with their arms by their sides, looking straight ahead. The level of body balance was assessed by the area of the 95th percentile ellipse (95 cm^2^ area), denoted as AVGCoP Area95^54^.

### Muscle strength assessment

Isometric knee muscle strength was measured using a Biodex System 4 dynamometer (Biodex Medical Systems, Inc., Shirley, NY, USA). Measurements of the peak torque were taken for the flexion and extension at the knee joint in the conditions of a 5-s isometric contraction. Each participant received an explanation and was familiarised with the test procedure the day before the muscle strength assessment by performing one set of submaximal contractions. After a 20-min standardized warm-up, the subjects were positioned on the equipment according to the manufacturer’s manual. All tests were conducted in a sitting position with the trunk and lower limbs stabilized with belts. The measurements of the knee torque were performed with an angular position of 90° in the knee and hip joints. During all measurements, the subject was given verbal encouragement to achieve their maximum potential. Each of the peak torque measurements for particular joints was made three times with 1-min breaks in between. The highest peak torques were used for analysis. Data collection was performed using a Compaq Desk Pro personal computer and the Biodex software following the standard Biodex protocol^55^.

### Statistical analysis

Two-way analysis of variance (ANOVA) was performed to examine the group and time main effects. If the ANOVA yielded a significant effect, a Bonferroni test was used for post hoc comparisons. The level of significance was set as p = 0.05 for all of the analyses. The normality of the data distribution was checked using the Shapiro–Wilk *W* test or Kolmogorov–Smirnov test. Changes (delta) in both groups were compared using a Mann–Whitney *U* test. The effect size (η^2^) was also calculated. The values of η^2^ were interpreted as follows: 0.1 is a small effect, 0.3 is a medium effect and 0.5 is a large effect, as described previously^29^. The required sample size was calculated a priori using G*Power 3.1.9.7. Meta-analytical findings reported small- to moderate effects of exercise on cognitive function in older people^4,8^. Considering a moderate effect (based on the previous studies), p = 0.05, 40 participants were required to reach 80% power on a repeated-measures ANOVA. All data are expressed as the mean ± standard deviation (SD) or standard error of the mean (SEM).

## Supplementary Information

Supplementary Information.

## Data Availability

Data may be available upon request by email to the principal investigator sylwester.kujach@gumed.edu.pl on reasonable request.
