# Supplementary material for: Judo training program improves brain and muscle function and elevates the peripheral BDNF concentration among the elderly
Source: Sci Rep. 2022 Aug 16;12:13900. doi: 10.1038/s41598-022-17719-6 (PMC9381784; doi:10.1038/s41598-022-17719-6)
Supplement: Supplementary file 1 — Supplementary Information. [file 41598_2022_17719_MOESM1_ESM.pdf]

Title: Judo training program improves brain and muscle function and elevates the peripheral BDNF concentration among the elderly

Sylwester Kujach[1,2*]., Maciej Chroboczek[2], Joanna Jaworska[3], Angelika Sawicka[4], Miroslaw Smaruj[5], Pawel Winklewski[1,6], Radoslaw Laskowski[2]

[1]Department of Human Physiology, Medical University of Gdańsk, Gdańsk, Poland.
[2]Department of Physiology, Gdansk University of Physical Education and Sport, Gdańsk, Poland.
[3] Department of Physiology, Medical University of Gdańsk, Gdańsk, Poland.
[4]Applied Cognitive Neuroscience Lab, Department of Human Physiology, Medical University of Gdańsk, Gdańsk, Poland.
[5]Department of Theory of Sport and Human Motorics, Gdansk University of Physical Education and Sport, Gdańsk, Poland.
[6]2nd Department of Radiology, Medical University of Gdańsk, Gdańsk, Poland.

Supplementary materials:

Detailed Training Intervention Description

1. Training intervention

The JEX subjects participated in 36 training sessions for 12 weeks, 3 times a week. Each training session lasted 45 minutes. The training program was based on selected exercises from the Kodokan Judo Institute in Tokyo. The training protocol was created by a judo master class trainer from 7th Dan. The technique exercises and the intensity of the efforts were adjusted to the capabilities and age of the participants. Training sessions were held in a judo training room (dojo) with specialist mattresses (tatami). The instructor informed the respondents about the rules of safety and hygiene behavior in judo classes. Participants received regulations and a glossary with the names of judo technical elements.

Each training session consists of 3 sections: A - warming up, B - the main training program, and C – cooling down. Figure 1. The training load increased with the following weeks of the experiment, and the redistribution of training parts (A, B, C) is highlighted in the supplementary material (Figure 1).

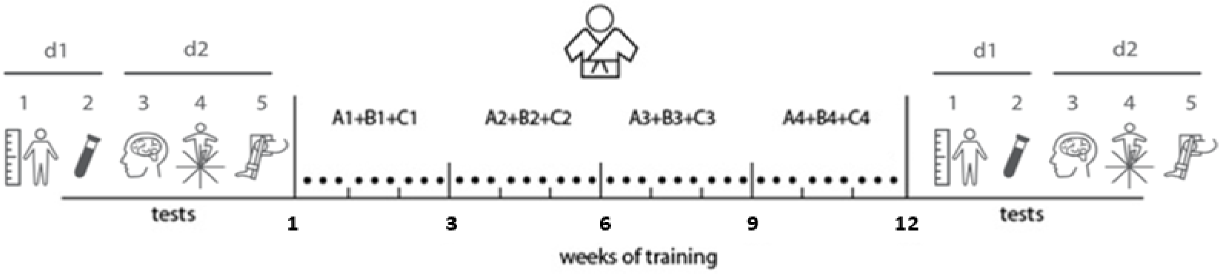

Fig1. The training load redistribution: 1-3 weeks A1,B1,C; 4-6 weeks A2,B2,C2; 7-9 weeks A3,B3,C3; 10-12 weeks A4,B4,C4.

Training protocol

A1: REI (teaching rules, decorum)

1.Walking around the tatami. 2.Flexibility and coordination exercises (duration around 5-10 minutes).

B1:

1. Teaching standing posture in judo (tachi shisei). 2. Teaching to move around tatami (shintai). 3. Teaching body rotation in judo (tai sabaki). 4. Teaching breakfall on tatami (ukemi). 5. Teaching judo techniques without a partner– solo practice (tandoku renshu): judo techniques on one leg without body rotation (osoto gari and deashi harai). 6. Teaching balance-breaking (kuzushi). 7. Teaching pinning techniques (osaekomi waza, honke kesa gatame, kami shiho gatame), (Duration around 30-35 minutes).

C1:

1. Mokuso, exercises relaxing in lying and standing positions. Collection-REI (duration around 5 minutes).

A2: REI

1. Walking around the tatami. 2. Flexibility and coordination exercises.

B2:

1. Improvement of standing posture in judo (tachi shisei). 2. Improving the movement of tatami (shintai).
3. Improvement of body rotation in judo (tai sabaki). 4. Teaching and improving breakfall on tatami (ukemi). 5. Teaching and disbudding judo techniques without a partner, individually (tandoku renshu). 6. Judo techniques on one leg without body rotation (osoto gari and deashi harai). 7. Perfecting balance-breaking (kuzushi). 8. Teaching and improving pinning techniques (osaekomi waza, yoko shiho gatame, honke kesa gatame, kami shiho gatame, tate shiho gatame). 9. Teaching repetition training (uchikomi, osoto gari). 10. Teaching with body rotation techniques (seoi nage). 11. Teaching combat-free mat work (ne waza - randori).

C2:

1. Mokuso, exercises relaxing in lying and standing. 2. Collection-REI

Exam: 6 kyu - white belt in judo

A3: REI

1. Walking around the tatami. 2. Flexibility and coordination exercises.

B3:

1. Improvement of standing posture in judo (tachi shisei). 2. Improving the movement of tatami (shintai).

3. Moving around tatami, judo body rotation (tai sabaki) 4. Tatami breakfall (ukemi). 5. Judo techniques without a partner, individually (tandoku renshu). 6. Perfecting balance-breaking (kuzushi). 7. Improvement of pinning techniques (osaekomi waza, yoko shiho gatame, honke kesa gatame, kami shiho gatame, tate shiho gatame). 8. Judo techniques on one leg without rotation (osoto gari, deashi barai). 9. Techniques with the body's rotation (seoi nage, o goshi). 10. Teaching repetition training (uchikomi). 11. Teaching combat-free mat work (ne waza randori).

All technique exercises were performed in series and on time, depending on the participants' capabilities.

C3:

1. Mokuso, exercises relaxing in lying and standing. 2. Collection REI

A4: REI

1. Walking around the tatami. 2. Flexibility and coordination exercises.

B4:

1. Perfecting standing posture in judo (tachi shisei). 2. Improving the movement of tatami (shintai). 3. Moving around tatami, body rotation in judo (tai sabaki). 4. Breakfall on tatami (ukemi). 5. Judo techniques without a partner, individually (tandoku renshu): osoto gari, deashi barai, seoi nage, o goshi - 3 sets of 10 times, a 1-minute break between the series. 6. Improving balance-breaking (kuzushi) - 3 sets of 10 times, break 1 minute between sets. 7. Improvement of pinning techniques (osaekomi waza, yoko shiho gatame, honke kesa gatame, kami shiho gatame, tate shiho gatame).

8. Repetition training (uchikomi) - 3 sets of 10 times, a 1-minute break between sets. 9. Teaching to throw (nagekomi) on the mattress: osoto gari. 10. Fights free mat work (ne waza randori) - 3 times for 2 minutes, break 3 minutes between fights.

C4:

1. Mokuso, exercises relaxing in lying and standing. 2. Collection REI

Exam: 5 kyu - yellow belt in judo

Names of judo techniques and translations were used according to the Kodokan New Japanese-English Dictionary of Judo, Kawamura and Daigo Kodokan (2001).

Table 1. Description and graphics of the judo exercises used in the training program.

**A1**	**Technique exercises**	**Description**<br>Kawamura and Daigo (2000)
REI<br>Decorum and decency and respect	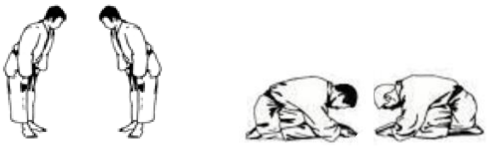<br>Ritsurei Zarei	An attitude of respect and consideration towards the partner or opponent and the forms of behavior expressing this attitude
1.Walking around the tatami	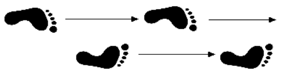	
2. Flexibility and coordination exercises	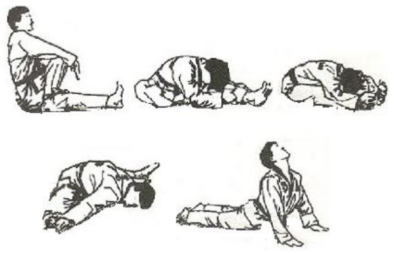	The special flexibility exercises used in judo
**B1**	**Technique exercises**	**Description**
1.Standing posture in judo (tachi shisei)	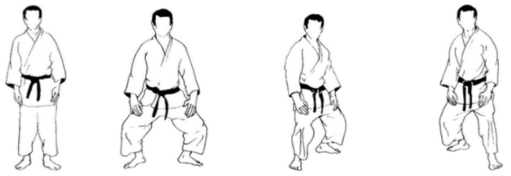<br>Natural posture (shizentai) · Defensive or ofensive posture (jigotai) · Right (migi jigotai) · Left (hidari jigotai)	Fighting stance, standing.
2. Move around tatami (shintai)	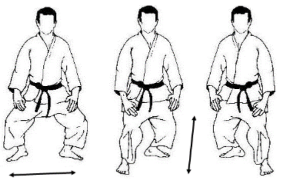	a) A method of movement in which the bottoms of the feet brush lightly over the surface of the mat, done to minimize vertical or lateral shifting of the center of gravity

	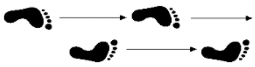 a) Slide stepping (suriashi) 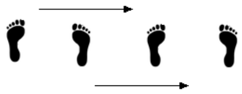 b) Shuffle stepping (tsugiashi)	b) A method of movement by stepping with one foot, drawing the other foot up to meet it, then stepping with the first foot again
3. Body rotation in judo (tai sabaki)	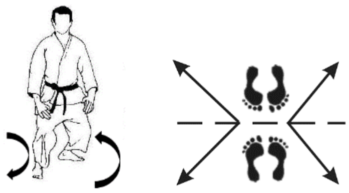	General term for movements used to shift the position of the body and change directions in the process of reacting to the opponent's techniques and setting up and applying own techniques.
4. Breakfall on tatami (ukemi)	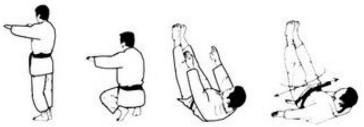 Breakfall backward (ushiro ukemi) 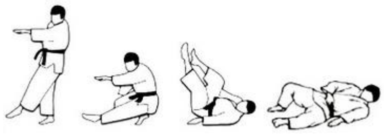 Breakfall aside (yoko ukemi) 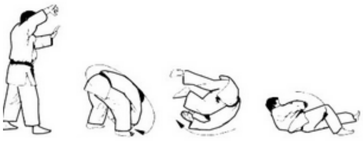 Forward rolling breakfall (mae mawari ukemi)	The general term for break falls (is designed to protect the body when thrown)
5. Individually – solo practice (tandoku renshu)	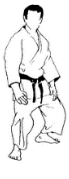	Solo practice using mirrors, etc. for the purpose of perfecting form and other aspects of one's own techniques
6.Balance-breaking (kuzushi)	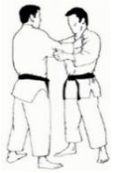 Engagement positioning, taking grips (kumikata, kumite)	An action to unbalance the opponent in preparation for a throw

	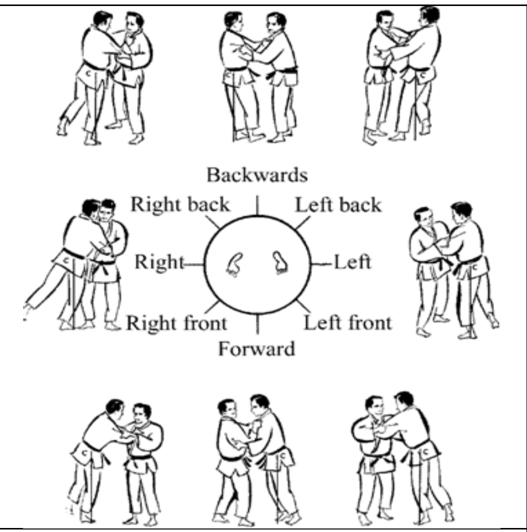	
7. Pinning techniques (osaekomi waza)	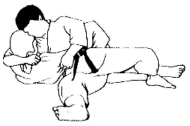<br>Scarf hold (honke kesa gatame)<br>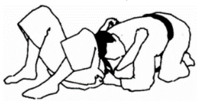<br>Upper four-corner hold (kami shiho gatame)	Mat techniques are used to pin a supine opponent to the mat, generally from the side or diagonally, to inhibit the freedom of movement and prevent from rising
**C1**	**Technique exercises**	**Description**
1. Mokuso, exercises relaxing in lying and standing position	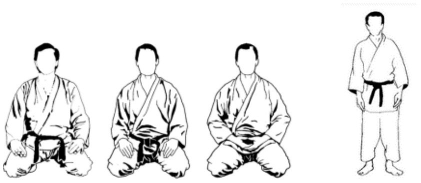	Mokuso is a Japanese term for meditation that means "looking silently into the heart" although it is also attributed to the translation of "calm reflection"
**A2**	see A1	
**B2**	**Technique exercises**	**Description**
1-7	see B1	
8. Pinning techniques (osaekomi waza)	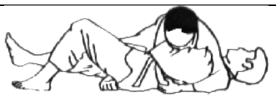<br>Side-locking four-corner hold<br>(yoko shiho gatame)<br>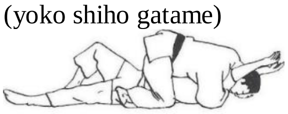<br>Straight four –corner hold<br>(tate shiho gatame)	see B1, 7
9. Teaching repetition training	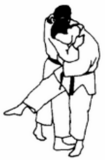<br>a)uchikomi	a) The repetitive application of a particular technique to learn the specific balance breaking, body shifting, power application, and other

	b)Large outer reap (osoto gari)	technical aspects associated with it<br>b) A foot techniques throw, break the opponent's balance to his rear or right rear corner to shift most of his weight onto his right heel, then reap his right leg with the right leg
10. Teaching with body rotation techniques (uchikomi)	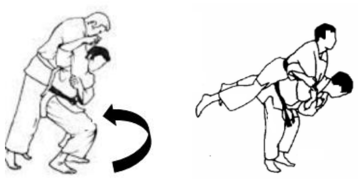<br>a) uchikomi<br>b)Shoulder throw (seoi nage)	a) see up<br>b) Throwing hand techniques break the opponent’s balance to his front or right front corner, then pivot to the left while allowing the elbow of your right arm to bend and come under his right armpit, then load him onto your back and throw him over your right shoulder
11. Teaching combat free mat work (ne waza randori)	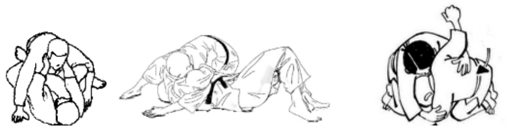	Practice sparring sessions in which both participants practice attacking and defending using freely applied throwing or/ and pining techniques<br>**We used in the study group grappling forms (only pining techniques)**
**C2**	see C1	
Exam: 6 kyu - white belt in judo	A test of the following techniques in judo: Rei, tachi shisei, shintai, ukemi, kuzushi, tai sabaki	Practitioners of Judo are ranked according to skill and knowledge of the sport. Their rank is indicated by the color of the belt that they wear.
**A3**	see A1, A2	
**B3**	**Technique exercises**	**Description**
1-7	see B2	

8. Judo techniques on one leg without rotation (uchikomi)	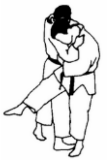 b) Large outer reap (osoto gari)<br>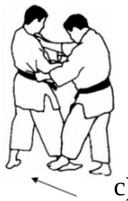 c) Forward foot sweep (de ashi harai)	a) uchikomi, see B2, 8<br>b) see B2, 9<br>c) A foot techniques, at the instant the opponent has taken a step forward or backward and is on the verge of placing his weight on the stepping foot, use your foot to sweep that foot from the rear, side or front.
9. Techniques with the rotation of the body (seoi nage, o goshi)<br><br>10. Teaching repetition training (uchikomi)	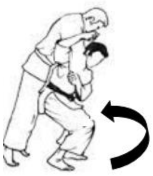 b) Shoulder throw (seoi nage)<br>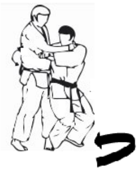 c) Large hip throw (ogoshi)	a) uchikomi see B2, 8<br>b) see B2, 9<br>c) A hip techniques throw, just as the opponent's balance to his front or right front corner, enter deeply with your right hip to float him up, and twist to throw him over it.
11. Teaching combat free mat work (ne waza randori)	see B2	
**C3**	see C1, C2	
**A4**	see A1, A2, A3	
**B4**	**Technique exercises**	**Description**
1-8	see B3, but 5-8 exercises with a certain amount of exercise time and rest breaks	Training<br>40-70% HRmax
9. Teaching to throw (nagekomi) on the mattress: osoto gari	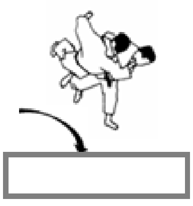	a) Throwing practice in which several opponents are throwing repeatedly using a predetermined technique for an agreed-upon number of repetitions or length of time,

	a) nagekomi, b) Large outer reap (osoto gari)	an important training method in developing throwing technique.<br>b) see B2, 9<br>**We used a specialized soft mattress for a safe fall**
10	see B3. 11	
**C4**	see C1, C2, C3	
Exam: 5 kyu - yellow belt in judo	A test of the following techniques in judo: Rei, osaekomi waza, tandoku renshu, uchikomi, nage komi	Practitioners of Judo are ranked according to skill and knowledge of the sport. Their rank is indicated by the color of the belt that they wear.

Glossary for participants (JAP/POL):

UKE – WSPÓŁĆWICZĄCY

TORI – OSOBA ĆWICZĄCA

REI - UKŁON/OKAZANIE SZACUNKU DRUGIEJ OSOBIE

ICH-1 NI-2 SAN-3 SHI-4 GO-5 ROKU-6 SHICHI-7 HACHI-8 KYU-9 JU(DŻU)-10

TSUGI ASHI – KROK PRZESTAWNY (PRAWO/LEWO, SKOS PRZÓD/SKOS TYŁ)

TSURI ASHI – KROK PRZESUWANY (PRZÓD/TYŁ)

TAI SABAKI – OBROTY CIAŁA

UKEMI – UPADKI/PADY

USHIRO UKEMI – PAD W TYŁ

YOKO UKEMI – PAD NA BOK

SHIZEN HONTAI – POSTAWA NATURALNA

JIGO HONTAI – POSTAWA OBRONNA/ GOTOWOŚĆ DO WALKI

MIGI – PRAWA

HIDARI – LEWA

TANDOKU RENCHU – ĆWICZENIA JUDO BEZ PARTNERA

OSOTO GARI – DUŻE ZEWNĘTRZNE PODCIĘCIE

OGOSHI – DUŻE BIODRO

DEASHI BARAI – ZAGARNIĘCIE NOGI WYSTAWIONEJ

OKURI ASHI BARAI – ZAGARNIĘCIE NOGI PRZESUWANEJ

SEOI NAGE – NOSIĆ NA PLECACH

KUZUSHI – WYCHYLENIE

UCHI KOMI – WEJŚCIA DO RZUTU Z PARTNEREM

HON KESA GATAME – PODSTAWOWE TRZYMANIE OPASUJĄCE

YOKO SHIHO GATAME – BOCZNE TRZYMANIE CZWOROBOCZNE

KAMI SHIHO GATAME – GÓRNE TRZYMANIE CZWOROBOCZNE

TATE SHIHO GATAME – RÓWNOLEGŁE TRZYMANIE CZWOROBOCZNE
